# Mercury: Tipping the Scales Toward Fish

**DOI:** 10.1289/ehp.115-a77a

**Published:** 2007-02

**Authors:** Adrian Burton

The benefits of eating moderate amounts of fish greatly outweigh the risks associated with intake of their potential contaminants, researchers report in the 18 October 2006 issue of *JAMA*. “People have heard that eating fish, especially oily fish, is good for them, yet they also hear that fish are often contaminated with mercury and other harmful pollutants,” explains lead author Dariush Mozaffarian, a professor in the Department of Epidemiology at the Harvard School of Public Health. “So what should one do? Our study compares the benefits and risks of fish consumption in order to provide an answer.”

The investigators searched the literature for studies evaluating the effects of fish or fish oil consumption on cardiovascular risk, mortality, and neurodevelopment, and the health risks associated with intake of methylmercury, polychlorinated biphenyls (PCBs), and dioxins from fish. Pooled and meta-analyses were then performed to better determine their associations.

The results indicated that modest consumption of fish or fish oil reduced the risk of death from heart attack by 36% and overall mortality by 17%, according to Mozaffarian. “For developing babies and infants, improved brain development seems to occur when mothers consume modest amounts of fish. Health benefits would be greatest from oily fish such as salmon, herring, or sardines; these have larger quantities of eicosapentaenoic acid and docosahexaenoic acid.” Many studies have linked these n-3 polyunsaturated fatty acids to such health benefits.

The researchers found that an average daily intake of 250 mg of eicosapentaenoic acid and docosahexaenoic acid, which can be gained from eating just one 3-oz portion of farmed salmon or one 6-oz portion of wild salmon per week, was sufficient for optimum protection against heart attacks. “Eating more is not necessary; there seems to be a threshold effect,” explains Mozaffarian.

The health risks associated with methylmercury (a bioactive form of mercury that accumulates in body fat and concentrates in the tissues of top predators) were less clear-cut. “Methylmercury has clear health risks when people are exposed to extremely high levels—for example, following industrial accidents,” says Mozaffarian. However, his analysis showed no clear health effects of lower levels of exposure in adults, such as seen with modest consumption of fish. This, he says, suggests the benefits of modest consumption outweigh the risks.

The same may be true for possible health effects of PCBs and dioxins. For example, the researchers report that eating wild or farmed salmon regularly over a lifetime could lead to 8 to 24 extra cancer deaths per 100,000 people, but more than 7,000 fewer cardiac deaths. They suggest, however, that women who might become pregnant should avoid eating too much shark, swordfish, or other fish with higher methylmercury levels, to reduce the risk of fetal neurodevelopmental problems.

“People who are allergic to fish or don’t like it could get their n-3 fatty acids via supplements or foods enriched with them,” says Rosa Ortega, a professor of nutrition at the Universidad Complutense in Madrid, Spain. “There are now many foods enriched in this way. However, it remains to be seen whether they provide the same benefits as whole fish.” She says people should also remember that cooking with used frying oil, as many restaurants do, can increase free radical damage in cells, “so fast-food fish-burgers may not be the best way to protect your heart.”

## Figures and Tables

**Figure:**
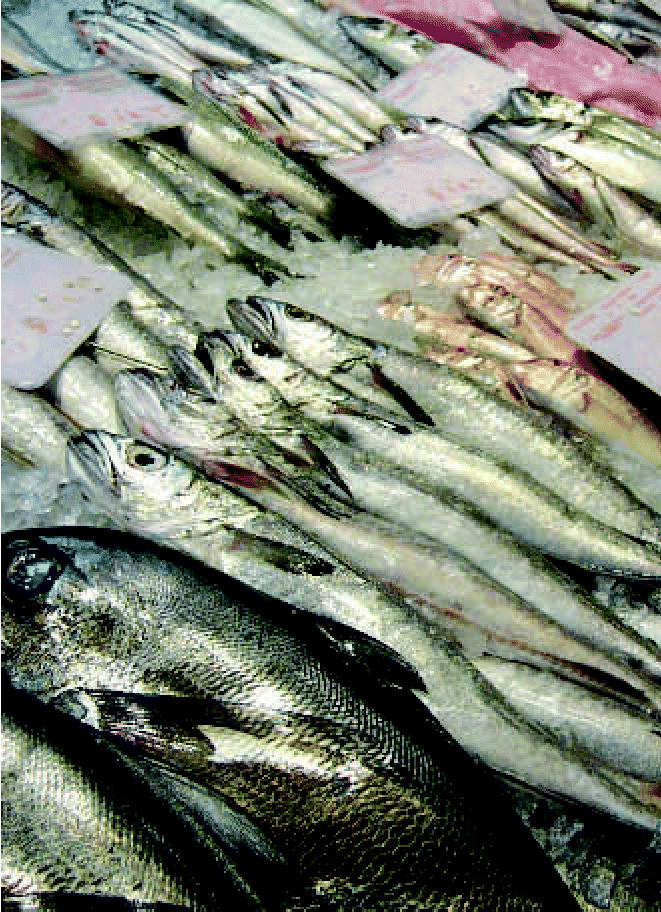
Health benefits are no fish tale A study comparing the benefits of eating fish with the risks of consuming environmental contaminants in this food found that moderate intake, for most people, is safe and more healthful than no fish at all.

